# A phase II study of the PI3K inhibitor copanlisib in combination with the anti-CD20 monoclonal antibody rituximab for patients with marginal zone lymphoma: treatment rationale and protocol design of the COUP-1 trial

**DOI:** 10.1186/s12885-021-08464-6

**Published:** 2021-06-29

**Authors:** Alexander Grunenberg, Lisa M. Kaiser, Stephanie Woelfle, Birgit Schmelzle, Andreas Viardot, Peter Möller, Thomas F. E. Barth, Rainer Muche, Jens Dreyhaupt, Markus Raderer, Barbara Kiesewetter, Christian Buske

**Affiliations:** 1 Department of Internal Medicine III, University Hospital Ulm, Albert-Einstein-Allee 23, 89081 Ulm, Germany; 2 Comprehensive Cancer Center Ulm, Institute of Experimental Cancer Research, University Hospital Ulm, Albert-Einstein-Allee 23, 89081 Ulm, Germany; 3 Institute of Pathology, Ulm University, Albert-Einstein-Allee 23, 89081 Ulm, Germany; 4 Institute of Epidemiology and Medical Biometry, Ulm University, Schwabstraße 13, 89081 Ulm, Germany; 5 Department of Medicine I, Division of Oncology, Medical University of Vienna, Vienna, Austria

**Keywords:** Marginal zone lymphoma, PI3K inhibitor, Copanlisib, Rituximab

## Abstract

**Background:**

Advanced stage marginal zone lymphoma (MZL) is an incurable indolent B-cell lymphoma, for which a wide variety of treatments ranging from single agent rituximab to more dose intense immunochemotherapy exists. One of the major goals in this palliative setting is to develop chemotherapy-free treatments, which approach the efficacy of immunochemotherapies, but avoid chemotherapy associated toxicity in this often elderly patient population. The PI3K inhibitor copanlisib has recently shown remarkable clinical activity in refractory or relapsed indolent B–cell lymphomas, among them MZL. Based on these data, copanlisib monotherapy was granted breakthrough designation by the FDA for the treatment of adult patients with relapsed marginal zone lymphoma who have received at least two prior therapies. However, data are still limited in particular for MZL. Based on this, the COUP-1 trial aims at testing the toxicity and efficacy of copanlisib in combination with rituximab in treatment naive and relapsed MZL.

**Methods:**

COUP-1 is a prospective, multicenter, single-arm, open-label, non-randomized phase II trial of 6 cycles (28 days cycle) of copanlisib (60 mg intravenous day 1, 8, 15) and rituximab (375 mg/m^2^ intravenous day 1) in the induction phase followed by a maintenance phase of copanlisib (d1, d15 every 4 weeks for a maximum of 12 cycles) and rituximab (d1 every 8 weeks for a maximum of 12 cycles) in patients aged ≥18 years with previously untreated or relapsed MZL in need of treatment. A total of 56 patients are to be enrolled. Primary endpoint is the complete response (CR) rate determined 12 months after start of induction therapy. Secondary endpoints include the overall response (OR) rate, progression free survival (PFS), overall survival (OS), safety and patient related outcome with quality of life.

The study includes a translational bio-sampling program with the prospect to measure minimal residual disease. The study was initiated in November 2019.

**Discussion:**

The COUP-1 trial evaluates the efficacy and toxicity of the treatment of copanlisib in combination with rituximab in patients with MZL and additionally offers the chance for translational research in this heterogenous type of lymphoma.

**Trial registration:**

ClinicalTrials.gov: NCT03474744. Registration date: 03/23/2018.

**Supplementary Information:**

The online version contains supplementary material available at 10.1186/s12885-021-08464-6.

## Background

### Marginal zone lymphoma

According to the WHO classification marginal zone lymphoma (MZL) is defined as a group of indolent non-Hodgkin lymphoma that accounts for about 10% of all B-cell lymphomas. MZL is a disease that includes three histological subtypes: splenic (SMZL), nodal (NMZL) and extranodal MZL (EMZL) with the latter tumor also known as mucosa-associated lymphoid tissue (MALT) lymphoma [1]. Although these subtypes share a similar immunophenotype, clinical and molecular characteristics as well as treatment approaches are distinct.

SMZL typically presents with enlargement of the spleen and bone marrow infiltration without or minimal peripheral lymphadenopathy. In SMZL, lymphocytosis is often an incidental finding, whereas anemia and thrombocytopenia are multifactorial features in advanced-stage disease due to bone marrow infiltration, hypersplenism and/or autoimmune phenomena. Definitive diagnosis of SMZL is based on splenectomy but is often made by combining diagnostic results of peripheral blood and bone marrow examination [2].

The clinical picture of NMZL often resembles the pattern of other nodal lymphomas, such as follicular lymphoma, and is characterized by the enlargement of peripheral, abdominal, and thoracic lymph nodes and is usually non-bulky. Diagnosis requires the exclusion of extranodal manifestations and splenic involvement [3].

The most common form is extranodal marginal zone lymphoma, which can occur in a large variety of different extranodal organs. It typically shows so-called lymphoepithelial lesions with epithelial invasion by clonal B cells and has only a low tendency to disseminate. MZL is often associated with either chronic infections as a result of chronic antigenic stimulation (e.g. Helicobacter pylori in gastric MZL lymphoma) or organ-related autoimmune diseases (e.g. Sjögren’s syndrome or Hashimoto’s thyreoiditis) [4].

### Therapeutic options for marginal zone lymphoma

Due to the indolent course of disease, an initial watch and wait strategy in the asymptomatic patient can be recommended. If the patient is in need of treatment, there are basically three options including anti-infective (antibiotics, antiviral medication), local (surgery, radiotherapy) and systemic treatment approaches. However, treatment algorithms are mainly based on retrospective data or extrapolated from studies of other lymphoma subtypes. Partly, because of the heterogeneity and the relative rarity of each MZL subtype, conducting studies remain a particular challenge in this disease.

### Rituximab as a therapeutic strategy for MZL

The CD20 antigen is expressed by B-lymphocytes on the surface of pre-B to the mature germinal center B-cells and the majority of mature B-cell neoplasia.

Rituximab targets specifically with high affinity (5 × 10^− 9^ mol/L) the glycolysated transmembrane phosphoprotein CD20. Upon binding of the Fab-fragment of the antibody to the CD20 antigen the Fc-fragment mediates various immunological responses (CDC = complement-dependent cytotoxicity, ADCC = antibody dependent cell-mediated cytotoxicity, ADP = antibody dependent phagocytosis) that contribute to the effective destruction of CD20 positive B-cells and thereby is a promising target in MZL [5–9]. Apart from the immune mediated mechanisms CD20 bound rituximab can initiate direct signaling induced cell death through redistribution of CD20 to lipid rafts (membrane domains rich in cholesterol and sphingolipids) that affect cell membrane and intracellular signaling effects [10].

Rituximab monotherapy is well tolerated, safe and has a reasonable anti-lymphoma activity at least in SMZL and EMZL. According to a recently published meta-analysis, assessing the efficacy of rituximab monotherapy in MZL, best response rates are achieved in SMZL [11] (see Table 1). Rituximab efficacy can be further augmented by adding chemotherapy. The largest trial, the IELSG-19 trial, randomized patients between chlorambucil, rituximab*/*chlorambucil and rituximab single agent in patients with no prior treatment of EMZL. In this trial, overall response (OR) rate was 78.3% with 55.8% complete response (CR) and 22.5% partial response (PR) for rituximab monotherapy compared with 94.7, 78.8 and 15.9%, respectively, for rituximab*/*chlorambucil [12, 13]. However, depending on the type of selected treatment approach, immunochemotherapy can be associated with considerable morbidity (grade ≥ 3 adverse events about 80%) and mortality rate (up to 11.9%) in MZL [14, 15].

**Table 1:** Efficacy data of rituximab monotherapy depending on MZL subtypes

MZL subtype		Number of patients	OR (%)	CR (%)
EMZL	[11]	112	73	45
	[12]	138	78	56
SMZL [11]		122	92	58
NMZL [11]		3	33	0

*MZL* marginal zone lymphoma, *EMZL* extranodal marginal zone lymphoma, *SMZL* splenic marginal zone lymphoma, *NMZL* nodal marginal zone lymphoma, *OR* overall response, *CR* complete response

### Copanlisib as a therapeutic strategy for MZL

**Fig. 1:**
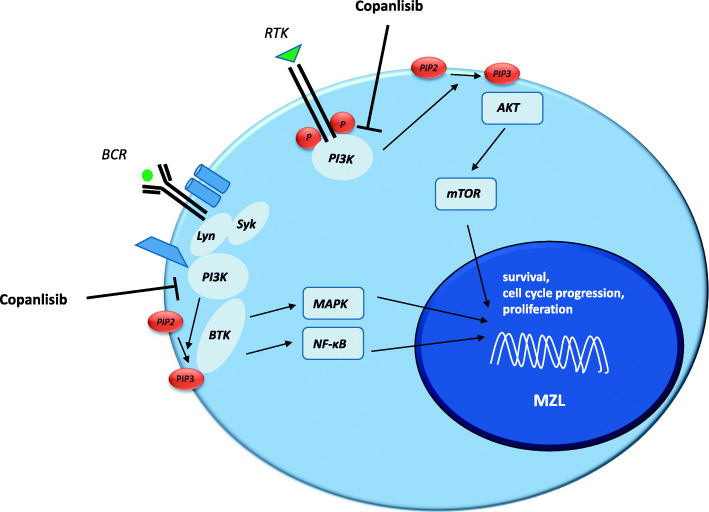
PI3K signaling pathway in physiological and malignant B-cells. PI3K activity is regulated in both a B-cell receptor (BCR) and receptor tyrosine kinase (RTK) mode. Upon RTK stimulation PI3K catalyses phosphatidylinositol 4,5-bisphosphonate (PIP2) to generate phosphatidylinositol 3,4,5-triphosphate (PIP3), resulting in recruitment of AKT (Protein kinase B) and activation of the mTOR (mechanistic target of rapamycin) pathway. On the other hand, BCR stimulation leads to the recruitment of BTK (bruton tyrosine kinase) via PIP3 with downstream activation of the MAPK (mitogen-activated protein kinase) and NFĸB (nuclear factor ‘kappa-light-chain-enhancer’ of activated B-cells) signaling pathway. Pathological triggering of both signaling pathways eventually results in increased proliferation and survival advantage of the B-cell. MZL = marginal zone lymphoma, Syk = spleen tyrosine kinase, Lyn = kinase

Nowadays, in the era of targeted medicine, small molecule inhibitors of kinases involved in B-cell signaling represent a promising area of research, including phosphatidylinositol-3-kinase (PI3K). PI3Ks are a family of heterodimeric kinases consisting of a catalytic and a regulatory subunit and are divided into three classes (class I, II and III). Four of these PI3K isoforms (PI3Kα, PI3Kβ, PI3Kγ, PI3Kδ) are categorized as class I enzymes because they can use phosphatidylinositol 4,5-bisphosphonate (PIP2) to generate phosphatidylinositol 3,4,5-triphosphate (PIP3). Elevated PIP3 in cellular membranes drives several hallmarks of the cancer phenotype such as proliferation, survival, and immune regulation (Fig. 1) [16]. PI3Kα and PI3Kβ are expressed ubiquitously, whereas PI3Kγ and PI3Kδ are highly restricted to hematopoietic tissue. Copanlisib (Bay 80–6946) is a potent pan-class I PI3K inhibitor, with approximately tenfold preferential inhibition of the PI3Kα and PI3Kδ isoform [17]. In 2016, a first-in-human phase I study reported promising anti-tumor activity of copanlisib, particularly in a small cohort of patients with advanced stage pretreated follicular lymphoma [18]. In line with that, Dreyling and colleagues demonstrated remarkable efficacy and manageable toxicity in heavily pretreated patients with indolent lymphoma. The OR was 43.7% in the indolent cohort [19]. Recently, the results of the pivotal phase II CHRONOS-1 study have led to the approval of copanlisib by the FDA. In this study, 142 patients with relapsed or refractory indolent lymphoma after two or more lines of therapy were enrolled, among 23 patients with MZL. In this lymphoma subtype the OR was 70%, including 9% with a CR and 61% with a PR (see Table 2) [21].

**Table 2:** Selected data of copanlisib monotherapy in relapsed or refractory B-cell-lymphoma subtypes in clinical trials

Trial	Subtype of lymphoma (%)	Number of patients	OR (%)	CR (%)	summary of treatment-emergent ≥ grade 3 (%) occurring in ≥10% of the total population
Phase I [18]	FL (66.6), DLBCL (33.3)	9	78%	11%	hyperglycemia (33), hypertension (33), lung infection (22), neutropenia (11), rash (11), anemia (11)
Phase II [20]	DLBCL (100)	79	19.4	5	hyperglycemia (33), hypertension (31)
Phase II [21]	FL (73), MZL (16), SLL 8 (6), LPL/WM (4), DLBCL (1)	142	59 (overall cohort), 70 (only MZL)	12 (overall cohort), 9 (only MZL)	hyperglycemia (41), hypertension (24), lung infection (15), neutropenia (24)

*FL* follicular lymphoma, *DLBCL* diffuse large B-cell lymphoma, *MZL* marginal zone lymphoma, *SLL* small lymphocytic lymphoma, *LPL* lymphoplasmacytic lymphoma, *WM* Waldenström’s macroglobulinemia, *OR* overall response, *CR* complete response

### Study rationale

So far, there is no well-established standard treatment for patients with MZL and systemic treatment approaches range from rituximab monotherapy to more intense immunochemotherapy. The dilemma here is that data do not consistently show an advantage on patient’s outcome with dose intense treatment. Moreover, it has to be taken into account that none of the dose intense approaches are regarded as curative and that the majority of patients suffering from MZL are elderly, highlighting the need to actively pursue safe and effective therapeutic options. Thus, mild chemotherapy-free approaches are highly attractive in this disease and the feasibility of this approach has been recently shown for the BTK inhibitor ibrutinib [22]. The hypothesis of this study is that adding copanlisib to rituximab is superior to rituximab monotherapy and is approaching the efficacy of immunochemotherapy without exposing the patient to chemotherapy associated toxicity.

## Methods and study design

The COUP-1 is a multicenter, open label, single-arm phase II study. The objective of the trial is to test the efficacy and toxicity of the treatment of the combination of copanlisib and rituximab in patients with MZL in need of treatment, who have failed or are not eligible for local therapy or relapsed after local or systemic treatment. For efficacy the rate of complete remissions according to the GELA criteria for gastric MZL or to Cheson 2007 criteria for non-gastric extranodal, nodal and splenic MZL after induction therapy will be primary analysed. The trial is registered on the clinicaltrials.gov database (NCT03474744) and is conducted in accordance with the Declaration of Helsinki. The ethics committee of Ulm University approved the COUP-1 trial as leading ethics committee for all German sites. In addition, the leading Austrian ethics committee also approved the study.

### Key inclusion and exclusion criteria

Eligible patients must be aged ≥18 years, be able to provide written informed consent and must have a proven pathological diagnosis of either extranodal, nodal or splenic CD20-positive MZL, confirmed by a reference pathology center. Patients have to be in need of treatment and both treatment naïve and relapsed/refractory patients can be included. Treatment naïve patients have to be not eligible for local treatment or local treatment must have been failed in these patients. In patients with SMZL without splenic tissue available for histologic review, the diagnosis may be confirmed by the presence of splenomegaly and typical morphologic and immunophenotypic findings in the blood and bone marrow. At least one bi-dimensionally measurable lesion (≥1.5 cm) in its largest dimension by CT scan or MRI must be met. Of note, for gastric MZL, the clinical evidence of the MZL as seen by gastroendoscopy is sufficient. All patients should have adequate bone marrow function (neutrophil counts ≥0.75 × 10^9^*/L* or baseline platelet count ≥50 × 10^9^*/L* [if not due to bone marrow infiltration by the lymphoma], GFR ≥ 40 mL/min/1.73m^2^, AST and ALT ≤3 × upper limit of normal and bilirubin ≤2 mg*/*dL or 2 × upper limit of normal).

Main exclusion criteria include ECOG *≥*2, CNS lymphoma, histologic evidence of transformation to a high grade or diffuse large B-cell lymphoma as well as chronic active hepatitis B. Ongoing immunosuppressive therapy including corticosteroids (exception *<* 4 weeks administered at a dose equivalent to ≤40 mg*/*day prednisone is allowed) are not permitted. Additional exclusion criteria comprise an unstable or new-onset angina pectoris, myocardial infarction less than 6 months prior to test drug, congestive heart failure > class II, HbA1c > 8.5% and uncontrolled arterial hypertension despite optimal medical management.

### Treatment

**Fig. 2:**
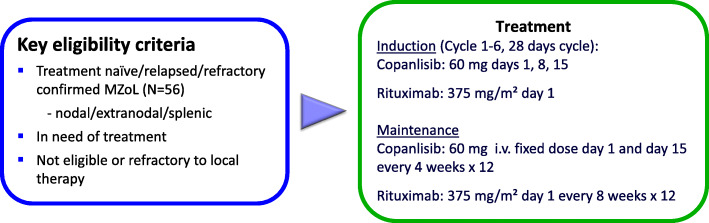
Trial design. i.v. = intravenous

Patient enrollment started in November 2019. Eligible patients receive an induction phase of copanlisib and rituximab followed by a maintenance phase of each test drug (Fig. 2). The induction phase consists of six cycles (1 cycle = 28 days). Copanlisib will be administered intravenously at a fix dose of 60 mg on days 1, 8, 15 of each cycle. Rituximab will be applied intravenously at a dose of 375 mg/m^2^ only on day 1 of each cycle.

Maintenance starts 2 months after the last induction cycle for patients at least achieving a stable disease after induction. During maintenance time copanlisib is administered at a dose of 60 mg on days 1 and 15 every 4 weeks for a maximum of 12 cycles. Rituximab application is scheduled at a dose of 375 mg/m^2^ every 8 weeks for a maximum of 12 infusions unless progression or study drug-related intolerable toxicity. In the follow-up phase, all subjects who enter the trial will continue to be followed every 3 months for disease progression, subsequent treatment and survival for 2 years after completion*/*discontinuation of treatment. Subsequently, patients will be monitored every 6 months for three additional years.

### Study objectives

The objective of the trial is to test the efficacy and toxicity of treatment with copanlisib and rituximab in patients with MZL in need of treatment, who have failed, are not eligible for local therapy or relapsed. The primary end point of this study is the CR rate determined 12 months after start of induction therapy. For efficacy the rate of complete remissions according to the GELA criteria for gastric MZL or to the Cheson 2007 criteria for non-gastric extranodal, nodal and splenic MZL will be primarily analysed. Secondary efficacy end points include response rates (CR, PR and OR [CR or PR]), best response, time to best response, time to first response, progression-free survival, time-to-treatment failure, remission duration, cause specific survival and overall survival. Quality of life assessment, based on the functional assessment of cancer therapy for lymphoma version 4 (FACTLym) will be measured before start of treatment, during induction and maintenance.

### Safety analyses

Safety variables include vital signs, physical examinations, evaluation of changes to concomitant medications, clinical laboratory parameters (hematology, serum chemistry), and the incidence, timing and severity of (serious) adverse events. The severity of adverse events will be graded using the CTCAE version 5.0 dictionary.

An independent external data safety monitoring committee (DSMC) will review ongoing safety data throughout the study. Review of the safety data by the DSMC will take place based on the safety analysis after the first 6 patients. In addition, a review will be performed when the 28th patient and the last patient has ended induction treatment. Following each meeting, the DSMC will prepare a report and may recommend changes in the conduct of the trial.

### Sample size calculation and statistical analysis

In MZL a variety of treatment modalities are used. So far rituximab single agent is the most frequent chemotherapy-free approach used in this entity. Thus, a novel chemotherapy-free approach should be at least as efficient as rituximab monotherapy. In a large randomized trial rituximab monotherapy induced a CR of 55.8% compared to 78.8% for the combination of immunochemotherapy in treatment naïve EMZL subjects. CR rates in splenic MZL are comparable high at about 60%, whereas CR rates in nodal MZL are reported a rare event. As the distribution of subtypes is about 70% EMZL, 20% splenic and 10% nodal MZL, a CR which is better than 56% should at least be achieved by a new chemotherapy-free approach at 12 months after start of therapy.

For sample size calculation the one-sided one sample exact binomial test was used. According to the above data, the CR for the total group of the different subtypes must be better than 56% 12 months after start of induction therapy. Based on a CR for copanlisib of about 75%, a significance level of 2.5% (because of one-sided test) and a power of 80%, 48 full evaluable patients will be necessary to show that the combination will be a promising candidate for challenging immunochemotherapy. It is expected, that the rate of withdrawal is smaller than 15%. According to these parameters, the study is planned to enroll 56 subjects. The distribution between the sexes is not relevant, because neither incidence of MZL differed between sexes nor clinically outcome measures have been shown to be related to sexes. The primary endpoint will be evaluated 12 months after the last patient recruited has started induction treatment. The primary parameter CR will be evaluated in a modified intention to treat way, which means that all patients for whom the primary endpoint CR is measured at 12 months after start of induction will be included in the analysis of the primary endpoint (core analysis population). This analysis population consists of all eligible patients included in the study who received at least one cycle of treatment. Patients without staging 12 months after start of treatment will be defined as non-responder (CR = ‘NO’). Patients who progress before end of induction therapy will be regarded as treatment failure. No primary end point will be determined for patients who withdraw. These patients will be excluded from the confirmatory data analysis but will be analyzed in a separate exploratory sensitivity analysis of the primary end point.

The decision about the new dual treatment concept of copanlisib in combination with rituximab will be based on the one-sided one sample exact binomial test, using CR ≤ 56% as H0. Thus, claim of success can be done if 36 (75% of 48 patients) or more CR will be observed. Additionally, a one-sided 97.5% confidence interval for CR will be calculated as an effect estimator. Exploratory use of univariate logistic regression models will be performed to investigate the influence of putative risk factors associated with CR. Subgroup analysis in the subtype strata will be performed as further exploratory analysis. All secondary end points will be analyzed exploratory by respective descriptive analysis and 95% confidence intervals.

### Biosampling program

**Fig. 3:**
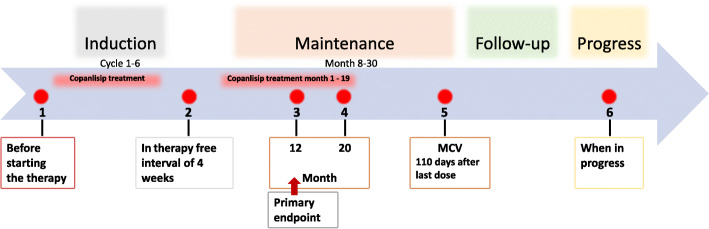
Timeline of biomarker analyses of the COUP-1 trial

The study includes a translational biosampling program which will serve as a platform for future research projects. The stored biospecimen program is a collection of serum, bone marrow and DNA specimens. Based on the study design, six eligible time points with sample collection prior to treatment, at the end of induction, at the primary endpoint (month 12), at the end of copanlisib treatment (month 20), at maintenance completion visit and in case of progression are defined (Fig. 3).

## Discussion

We describe here an open-label, multicentric phase II trial to confirm the efficacy and safety of the combination of rituximab with copanlisib in MZL patients, who are either treatment naive or have relapsed/refractory disease. In this mostly elderly patient population, the development of chemotherapy-free approaches is of particular interest. However, complete response rates shown with target molecules given as a single agent are usual low and might be explained to the activation of additional pathways in resistant cases [22]. Thereby there is an urgent need to test combinations of compounds with complementary mode of action, ensuring long-lasting eradication of MZL cells. Indeed, combination testing deciphered several compounds, among them rituximab, that synergize with copanlisib in B-cell lymphoma models [23].

The PI3Kδ inhibitor idelalisib was the first in its class to receive FDA approval in relapsed follicular lymphoma based on an overall response rate of 57% (CR 7%) in double refractory patients. Similar results were reported for relapsed MZL patients (OR 47%, no CR) [24]. However, early enthusiasm for this agent went down due to its toxicity profile and the drug subsequently gained a black box warning for adverse events such as hepatotoxicity, colitis, pneumonitis, and increased risk of opportunistic infections. Unexpected aggravation of adverse events in combined applications of idelalisib, including rituximab, even culminated in premature termination of trials and subsequently resulted in a restriction of its use despite the release of recommendations for the management of toxicity associated with idelalisib [25–27]. Copanlisib shows higher potency against all four PI3K class I isoforms and a more preferential targeting of the PI3Kα compared to idelalisib and actually seems to have a more favorable safety profile, raising the possibility that side effects of selective PI3Kδ inhibition might be avoided. The reported main side effects of copanlisib are mostly infusion-related including transient hyperglycemia, which is an expected on-target effect of PI3Kα inhibition that is related to insulin-receptor signaling [28, 29]. Besides hyperglycemia, most common reported higher treatment-related adverse events are transient hypertension and neutropenia. Regarding the unique side effect profile of copanlisib, recommendations on optimal management of copanlisib associated adverse events have been recently published and will help to manage safe drug administration [30].

Based on the known activity and the favorable toxicity profile of rituximab as single agent and the promising efficacy results of copanlisib, the concept of this chemotherapy-free treatment approach is highly attractive and will help to define the role of this combination in MZL patients, aiming at challenging immmunochemotherapy. It will further help to evaluate the feasibility of rituximab/copanlisib maintenance therapy after induction in patients with MZL.

## Supplementary Information

**Additional file 1.** SPIRIT (Standard Protocol Items: Recommendations for Interventional Trials) 2013 Checklist: Recommended items to address in a clinical trial protocol and related documents.

**Additional file 2.** Schedule of enrolment, interventions, and assessments.

## Data Availability

Not applicable.

## Abbreviations

ADCC: Antibody dependent cell-mediated cytotoxicity
ADP: Antibody dependent phagocytosis
BTK: Bruton tyrosine kinase
CD: Cluster of differentiation
CDC: Complement-dependent cytotoxicity
CNS: Central nervous system
CR: Complete response
CT: Computed tomography
CTCAE: Common Terminology Criteria for Adverse Events
DSMC: Data Safety Monitoring Committee
ECOG: Eastern Cooperative Oncology Group
EMZL: Extranodal marginal zone lymphoma
Fab: Fragment antigen-binding
FACT-Lym: Functional Assessment of Cancer Treatment-Lymphoma
Fc: Fragment crystallizable
FDA: Food and Drug Administration
GELA: Groupe d'Etude des Lymphomes de l'Adulte
GFR: Glomerular filtration rate
HbA1c: Hemoglobin A1c
H0: Null hypothesis
IELSG: International Extranodal Lymphoma Study Group
NMZL: Nodal marginal zone lymphoma
MRI: Magnetic resonance imaging
MZL: Marginal zone lymphoma
OR: Overall response
OS: Overall survival
PIP2: Phosphatidylinositol 4,5-bisphosphonate
PIP3: Phosphatidylinositol 3,4,5-bisphosphonate
PI3K: Phosphatidylinositol-3-kinase
PFS: Progression free survival
PR: Partial response
SMZL: Splenic marginal zone lymphoma
WHO: World Health Organization
