# Supplementary material for: A phase II study of the PI3K inhibitor copanlisib in combination with the anti-CD20 monoclonal antibody rituximab for patients with marginal zone lymphoma: treatment rationale and protocol design of the COUP-1 trial
Source: BMC Cancer. 2021 Jun 29;21:749. doi: 10.1186/s12885-021-08464-6 (PMC8243426; doi:10.1186/s12885-021-08464-6)
Supplement: Supplementary file 1 — Additional file 1. SPIRIT (Standard Protocol Items: Recommendations for Interventional Trials) 2013 Checklist: Recommended items to address in a clinical trial protocol and related documents. [file 12885_2021_8464_MOESM1_ESM.docx]

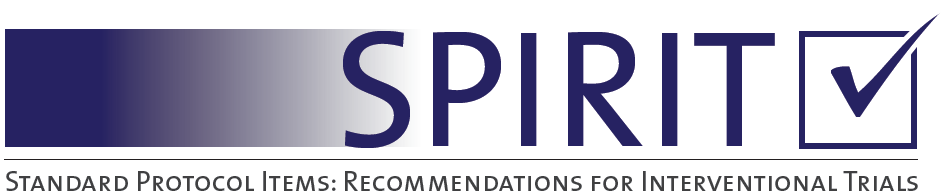


SPIRIT 2013 Checklist: Recommended items to address in a clinical trial protocol and related documents*

| Section/item | Item No | Description | Addressed on page number |
| --- | --- | --- | --- |
| **Administrative information** | | |  |
| Title | 1 | Copanlisib and Rituximab in Marginal Zone Lymphoma Patients. A Multicenter Open Label Single-Arm Phase II Study (COUP-1) | 1 |
| Trial registration | 2a | COUP-1  EudraCT: 2017-003150-16  ClinicalTrials (NCT): NCT03474744 | 3 |
|  | 2b | N/A |  |
| Protocol version | 3 | 4.0 (05.05.2020) | 15 |
| Funding | 4 | The trial is partially supported by Bayer AG. Support includes the supply of the study medication. Also Celltrion Healthcare supplies study medication. There is no other funding of the trial. The authors have no other financial relationship with Bayer AG or Celltrion Healthcare. | 14-15 |
| Roles and responsibilities | 5a | **Authors:**  Alexander Grunenberg^1^, Lisa M Kaiser^2^, Stephanie Wölfle^2^, Birgit Schmelzle^2^, Andreas Viardot^1^, Peter Möller^3^, Thomas FE Barth^3^, Rainer Muche^4^, Jens Dreyhaupt^4^, Markus Raderer^5^, Barbara Kiesewetter^5^, Christian Buske^1^  **Affiliations:**  ^1^Department of Internal Medicine III, University Hospital Ulm, Ulm, Germany  ^2^Comprehensive Cancer Center Ulm, Institute of Experimental Cancer Research, University Hospital Ulm, Ulm, Germany  ^3^Institute of Pathology, Ulm University, Ulm, Germany  ^4^Institute of Epidemiology & Medical Biometry, Ulm University, Ulm, Germany  ^5^ Department of Medicine I, Division of Oncology, Medical University of Vienna, Vienna, Austria. | 1 |
|  | 5b | University Hospital Ulm, Ulm, Germany; represented by the Chairman of the board.  Coordinating Investigator and contact for the sponsor: Prof. Dr. Christian Buske, University Hospital of Ulm, Department of Internal Medicine III, Albert-Einstein-Allee 23, 89081 Ulm, Germany. | 14 |
|  | 5c | The funding source and sponsor’s representative have no role in study design, data collection, data analysis, data interpretation, or writing of the report. | 14-15 |
|  | 5d | AG, CB, AV, MR and BK developed and planned this trial and CB is the principle investigator of the study. AG performed basic research and wrote the manuscript. PM and TFE Barth are conducting for reference pathology and diagnosis analysis. LMK coordinates an additional scientific program. RM and JD are the trial statistician and responsible for statistical planning and statistical analysis. SW and BS contributed in the trial design and modifications and data collection. All authors read and approved the final manuscript | 13 |
| Introduction |  |  |  |
| Background and rationale | 6a, 6b | For marginal zone lymphoma (MZL) Rituximab in combination with conventional chemotherapy is widely used for those patients who fail local therapy or do not qualify for such. Depending on the MZL subtype Rituximab/chemotherapy is able to induce in part long remissions, but do not prevent relapse later on. In addition, chemotherapy associated toxicity is often problematic in MZL patients, who are mostly of advanced age. Thus, chemotherapy–free approaches are highly attractive for this patient group. Rituximab single agent is a widely used chemotherapy–free approach in MZL, but was significantly inferior compared to Rituximab/Chlorambucil in a large randomized prospective clinical trial in treatment naïve MZL with a CR rate of 55.8% vs. 78.8%, respectively (P<0.001). Thus, it is the major aim to develop chemotherapy–free approaches for MZL, which approach efficacy of Rituximab/chemotherapy combinations, but avoid chemotherapy associated toxicities. This in particular important in MZL as many physicians are reluctant to treat these often elderly patients with more intense treatments and prefer single agent therapies in these very often well and long responding lymphoma subtype. The PI3K inhibitor Copanlisib has shown high clinical activity in indolent B–cell lymphomas among them MZL. Based on these observations it is the aim of this study to test the toxicity and efficacy of Copanlisib in combination with the anti-CD20 antibody Rituximab in patients with newly diagnosed or relapsed MZL in need of treatment, who are not eligible or failed local therapy, following the assumption that this novel chemotherapy–free combination is significantly more effective than Rituximab single agent therapy and at least as efficient as Rituximab/chemotherapy, but avoids chemotherapy–related toxicity. | 4-7 |
| Objectives | 7 | The objective of the trial is to test the efficacy and toxicity of the treatment of Copanlisib/Rituximab in patients with MZL in need of treatment, who have failed or are not eligible for local therapy or relapsed after local or systemic therapy. For efficacy the rate of complete remissions (according to the GELA criteria for gastric MALT or to the Cheson 2007 criteria for non-gastric extranodal, nodal and splenic MZL) after induction therapy will be primarily analysed. For toxicity treatment associated adverse events, quality of life and cumulative incidence of secondary malignancies will be documented. | 9 |
| Trial design | 8 | This study is a multicenter, single-arm, open-label, non-randomized phase II trial of 6 cycles of Copanlisib and Rituximab in the induction phase followed by a maintenance phase for a maximum of 12 infusions of Copanlisib every 4 weeks and Rituximab every 8 weeks in MZL patients aged ≥ 18 years in need of treatment. | 8-9 |
| Methods: Participants, interventions, and outcomes | | |  |
| Study setting | 9 | It is expected that a total of 56 patients at approximately 20 investigator sites in Germany and 1 center in Austria will be registered.  Access and availability of services are equal between the different sites. Procedures (e.g. drug applications, radiation therapy) have been defined as standard procedures as explained in the study protocol. All sites have been trained before start of the trial and are supervised in periodic intervals. | 7-11 |
| Eligibility criteria | 10 | Inclusion Criteria  Patients must meet all of the following inclusion criteria to be eligible for participation in this study:   - Confirmed CD20 positive MALT Lymphoma de novo following or being not eligible for local therapy (including surgery, radiotherapy) and antibiotics for H. pylori-positive gastric lymphoma arisen at any extranodal site or relapsed   OR   - Confirmed CD20 positive de novo following or not being eligible for local therapy (including surgery and antiviral therapy for Hepatitis C Virus) with symptomatic disease or relapsed splenic MZL   OR   - Confirmed CD20 positive de novo or relapsed nodal MZL   **For nodal and extragastric MALT lymphoma:**   - At least one bi-dimensionally measurable lesion (≥ 1.5 cm in its largest dimension by CT scan or MRI).   **For SMZL:**  For splenic MZL, an enlarged spleen on CT scan and lymphoma cell infiltration has to be seen in bone marrow and/or peripheral blood.  At least one of the following criteria must be met:   - Bulky progressive or painful splenomegaly - one of the following symptomatic/progressive cytopenias: Hb < 10 g/dL, or Plat < 80.000 /µL, or neutropenia < 1000/µL, whatever the reason (autoimmune or hypersplenism or bone marrow infiltration) - SMZL with concomitant hepatitis C infection who have not responded to or are relapsed after Interferon and/or Ribavirin (patients positive for HCV antibody are eligible only if PCR is negative for HCV RNA). - splenectomised patients with rapidly raising lymphocyte counts, development of lymphadenopathy or involvement of extranodal sites if not being eligible for local therapy   **For gastric MALT lymphoma:**   - H. pylori-negative and positive cases following or being not eligible for local therapy (i.e., surgery, radiotherapy or antibiotics) or after systemic therapy. - H. pylori-negative and positive disease that has remained stable, progressed or relapsed following antibiotic therapy - For gastric MALT lymphoma, the clinical evidence of the MZL as seen by gastroendoscopy is sufficient. There is no need to show a measurable lesion by CT scan or MRI. - Age ≥ 18 years - Life expectancy >3 months. - Baseline platelet count ≥ 50 ×10^9^/L (if not due to BM infiltration by the lymphoma), absolute neutrophil count ≥ 0.75×10^9^/L. - Meet the following pretreatment laboratory criteria at the Screening visit conducted within 28 days of study enrollment (unless due to underlying lymphoma):   - ASAT (SGOT): ≤3 times the upper limit of institutional laboratory normal value   - ALAT (SGPT): ≤3 times the upper limit of institutional laboratory normal value   - Total Bilirubin: ≤2 mg/dL or 2 times the upper limit of institutional laboratory normal value, unless clearly related to the disease (except if due to Gilbert’s syndrome) - GFR ≥ 40 mL/min/1.73 m² - Negative HIV antibody - Positive test results for chronic HBV infection (defined as positive HBsAg serology): patients with occult or prior HBV infection (defined as negative HBsAg and positive total HBcAb) may be included if HBV DNA is undetectable, provided that they are willing to undergo monthly DNA testing. Patients who have protective titers of HBsAb after vaccination or prior but cured hepatitis B are eligible. - Positive test results for hepatitis C (hepatitis C virus [HCV] antibody serology testing): patients positive for HCV antibody are eligible only if PCR is negative for HCV RNA. - Pregnancy β-HCG negative. For women of child-bearing potential only (i.e. fertile, following menarche and until becoming post-menopausal unless permanently sterile. Permanent sterilisation methods include hysterectomy, bilateral salpingectomy and bilateral oophorectomy); serum or urine β-HCG must be negative during screening and at study enrolment visit - Premenopausal fertile females must agree to use a highly effective method of birth control for the duration of the therapy up to 12 months after end of therapy. - Men must agree not to father a child for the duration of therapy and 6 months after (use of a condom) and must agree to advice a female partner to use a highly effective method of birth control. - Willingness and ability to comply with scheduled visits, drug administration plan, imaging studies, laboratory tests, other study procedures, and study restrictions. - Evidence of a personally signed informed consent indicating that the subject is aware of the neoplastic nature of the disease and has been informed of the procedures to be followed, the experimental nature of the therapy, alternatives, potential benefits, possible side effects, potential risks and discomforts, and other pertinent aspects of study participation.   Exclusion Criteria  The presence of any of the following will exclude a subject from enrolment:   - ECOG performance status ≥ 2 - History of a non-lymphoid malignancy except for the following: adequately treated local basal cell or squamous cell carcinoma of the skin, cervical carcinoma in situ, superficial bladder cancer, asymptomatic prostate cancer without known metastatic disease and with no requirement for therapy or requiring only hormonal therapy and with normal prostate specific antigen for ≥1 year prior to study enrollment visit, other Stage 1 or 2 cancer treated with a curative intent and currently in complete remission, for ≥3 years. - Central nervous system lymphoma, leptomeningeal lymphoma, or histologic evidence of transformation to a high-grade or diffuse large B-cell lymphoma. - Ongoing immunosuppressive therapy including corticosteroids (exception < 4 weeks administered at a dose equivalent to ≤ 40 mg/day prednisone is allowed) - Evidence of ongoing systemic bacterial, fungal, or viral infection at the time of study enrolment visit - Ongoing drug-induced liver injury, chronic active hepatitis B (HBV), alcoholic liver disease, non-alcoholic steatohepatitis, primary biliary cholangitis, extrahepatic obstruction caused by cholelithiasis, cirrhosis of the liver, or portal hypertension. - Ongoing alcohol or drug addiction - Treatment with any other investigational agent within 30 days or within 5 x the half-life (t1/2) of the investigational product, whichever is longer, or participating in another trial within 30 days prior to entering this study - Breastfeeding or pregnancy - Prior treatment with Copanlisib - Congestive heart failure > New York Heart Association (NYHA) class 2 - Unstable angina (angina symptoms at rest), new-onset angina (begun within the last 3 months). - Myocardial infarction less than 6 months before start of test drug - Uncontrolled arterial hypertension despite optimal medical management - HbA1c>8.5% - Prior or ongoing clinically significant illness, medical condition, surgical history, physical finding, electrocardiogram (ECG) finding, or laboratory abnormality that, in the investigator’s opinion, could adversely affect the safety of the subject or impair the assessment of study results. - History of anaphylaxis in association with previous administration of monoclonal antibodies. - Vaccination with a live vaccine within 28 days prior to start of therapy - Arterial or venous thrombotic or embolic events such as cerebrovascular accident (including transient ischemic attacks), deep vein thrombosis or pulmonary embolism within 3 months before the start of study medication - Non-healing wound, ulcer, or bone fracture - History or concurrent interstitial lung disease of any severity and/or severely impaired lung function (as judged by the investigator). | 7-8 |
| Interventions | 11a | **Induction**  Cycle 1-6 (28 days cycle):   - Copanlisib: 60 mg i.v. fixed dose days 1, 8, 15. - Rituximab: 375 mg/m2 Rituximab i.v. day 1   Start of treatment with Copanlisib infusion followed by Rituximab infusion is recommended because glucose measurements after end of copanlisib infusion are required up to 1 and 2 hours after end of Copanlisib infusion.  **Maintenance**  Start 2 months after start of the last induction cycle (day 1) for patients at least achieving a stable response after induction.   - Copanlisib: 60 mg i.v. Copanlisib fixed dose day 1 and day 15 every 4 weeks for a maximum of 12 cycles (12 months) or until progression or study drug-related intolerable toxicity (month 2 to month 13 after end of induction) - Rituximab: 375 mg/m2 Rituximab i.v. day 1 every 8 weeks for a maximum of 12 infusions or until progression or study drug-related intolerable toxicity (month 2 to month 24 after end of induction)   Start of treatment with Copanlisib infusion followed by Rituximab infusion is recommended because glucose measurements after end of copanlisib infusion are required up to 1 hour after end of Copanlisib infusion.  **Follow-up Phase**  All subjects who enter the trial will continue to be followed every 3 months for disease progression, subsequent treatment, and survival for two years after completion/discontinuation of treatment. Subsequently, patients will be monitored every 6 months for three additional years.  See figure 1 below | 8-9 |
|  | 11b | For **Copanlisib**, in Phase I and II trials, certain toxicities were seen only in relation to Copanlisib e.g., transient increases in glucose and blood pressure. Based on this knowledge the investigator may decide on the necessary dose modifications.  Detailed guidelines are given for dose modifications and treatment interruptions for Copanlisib in hematological toxicity and non-hematological toxicity. As the instructions are very large they might be provided separately if requested.  However, dose modification levels of Copanlisib are outlined in Table 1:   \| Table 1: Dose levels of Copanlisib \| \| \| --- \| --- \| \| Dose level 1 (starting dose): \| 60 mg of Copanlisib \| \| Dose level -1: \| 45 mg of Copanlisib \| \| Dose level -2: \| 30 mg of Copanlisib \|   After having fully recovered from toxicity and in the absence of any criteria for further dose reduction or study drug discontinuation, re-escalation from dose level -2 to dose level -1, or from dose level -1 to dose level 1 will be allowed at the investigator’s discretion, with the exception of non-infectious pneumonitis (NIP). Patients who do not tolerate the Copanlisib dose of 30 mg must discontinue study treatment permanently.  For **Rituximab**, if any grade 1 or 2 IRR occurs during an infusion, infusion should be slowed or interrupted (at the discretion of the investigator) and supportive treatment instituted. The infusion rate can be increased or restarted on resolution of the symptoms.  If any grade 3 or 4 IRR occurs during an infusion, infusion will be discontinued immediately and not restarted until resolution of the symptoms. Treatment can be reinitiated (at half the original infusion rate) at the discretion of the investigator, but if the same adverse event appears again with the same severity, treatment must be permanently discontinued.  If bronchospasm or dyspnea occurs in the patient during the infusion, the infusion should be stopped immediately. Medication such as anti-histamines or steroids should be given for symptomatic relief. The infusion should not be re-started until symptoms have resolved completely and should be given at half the original infusion rate. Neutropenia and thrombocytopenia could be due to the course of the disease and it may be necessary to delay the dose of Rituximab until they are resolved, up to a maximum of 2 weeks. | 11-12 |
|  | 11c | Bayer AG and Celltrion Healthcare will provide the quantity of trial medication required for the clinical trial. The medication provided must be used only in the context of this clinical trial. Careful records will be kept of the trial medication supplied to the study sites. At the end of the study, all unused medication will be returned to manufacturer. If deficiencies of the trial medication are noticed, the monitor, the project manager and the LKP must be informed immediately. The order and delivery of the mediation includes the local pharmacy of each site. The order form for up to 6 applications of Rituximab for each patient will be sent by the study sites to Celltrion Healthcare. The order form for a box 24 vials of Copanlisib will be sent by the study sites to the Clinical Trials Office at the sponsor where the order is approved and sponsor’s pharmacy will provide sites with the medication. Monitoring will be done as on-site visits or remotely by a clinical monitor according to a Monitoring Plan. The monitor will review the entries into the eCRF on the basis of source documents. The investigator must allow the monitor to verify all essential documents and must provide support at all times to the monitor. The monitor will document the visit in a report for the sponsor. The site will be provided with a follow-up letter of the findings and the necessary actions to be taken. By frequent communications (letters, telephone, e-mail), the site monitor will ensure that the trial is conducted according to the protocol and regulatory requirements. Frequency and details of monitoring will be defined in the Monitoring Plan. If there are major findings during monitoring or an audit, the investigational site might be closed by the LKP. | 14 |
|  | 11d | see also 10.  Relevant additional diseases present at the time of informed consent are regarded as concomitant diseases and will be documented on the appropriate pages of the eCRF. Included are conditions that are seasonal, cyclic, or intermittent (e.g. seasonal allergies; intermittent headache). Abnormalities, which appear for the first time or worsen (intensity, frequency) during the trial are adverse events (AEs) and must be documented on the appropriate pages of the eCRF.  Prior and Concomitant Medication: The treatment of accompanying illnesses not subject to the exclusion criteria is permissible if this is not expected to have any effect on the outcome measures used in this study and to interfere with the trial medication. If concomitant drugs are administered, these must be recorded in the patient file and in the eCRF. A guiding list of permitted and prohibited concomitant medication is included in the study protocol. | 7-8 |
| Outcomes | 12 | Primary endpoint is the complete response (CR rate (CRR) determined 12 months after start of induction therapy, i.e. month 6 of maintenance).  Secondary endpoints are:   - Response rates (complete response (CR), partial response (PR)) and overall response rate (CR or PR)) evaluated 4 weeks after the end of induction treatment and 12 months after start of treatment. - Best response determined in the time interval from the start of induction therapy to end of follow-up. - Time to best response defined as the time from the start of induction to best response the patient achieves (CR, PR). - Time to first response defined as the time from the start of induction to first response (CR, PR). - Progression free survival (PFS) defined as the time from registration to the first occurrence of progression or relapse as assessed by the investigator, or death from any cause. - Time to treatment failure (TTF) defined as the time of registration to discontinuation of therapy for any reason including death from any cause, progression, toxicity or add-on of new anti-cancer therapy. - Duration of Response (DR) defined as the period from the first response (at least PR) to treatment until evidence of disease progression, relapse or death of any cause. - Cause specific survival (CSS) defined as the period from the induction registration to death from lymphoma or lymphoma related cause; death unrelated to MZL is considered as a competing event. - Overall survival (OS) defined as the period from the induction registration to death from any cause. - Quality of life measured by the FACT-Lym before start of treatment, during induction and maintenance therapy.   Safety variables will include AEs, SAEs, laboratory parameters, ECG and vital signs. | 9 |
| Participant timeline | 13 | see Fig. 2  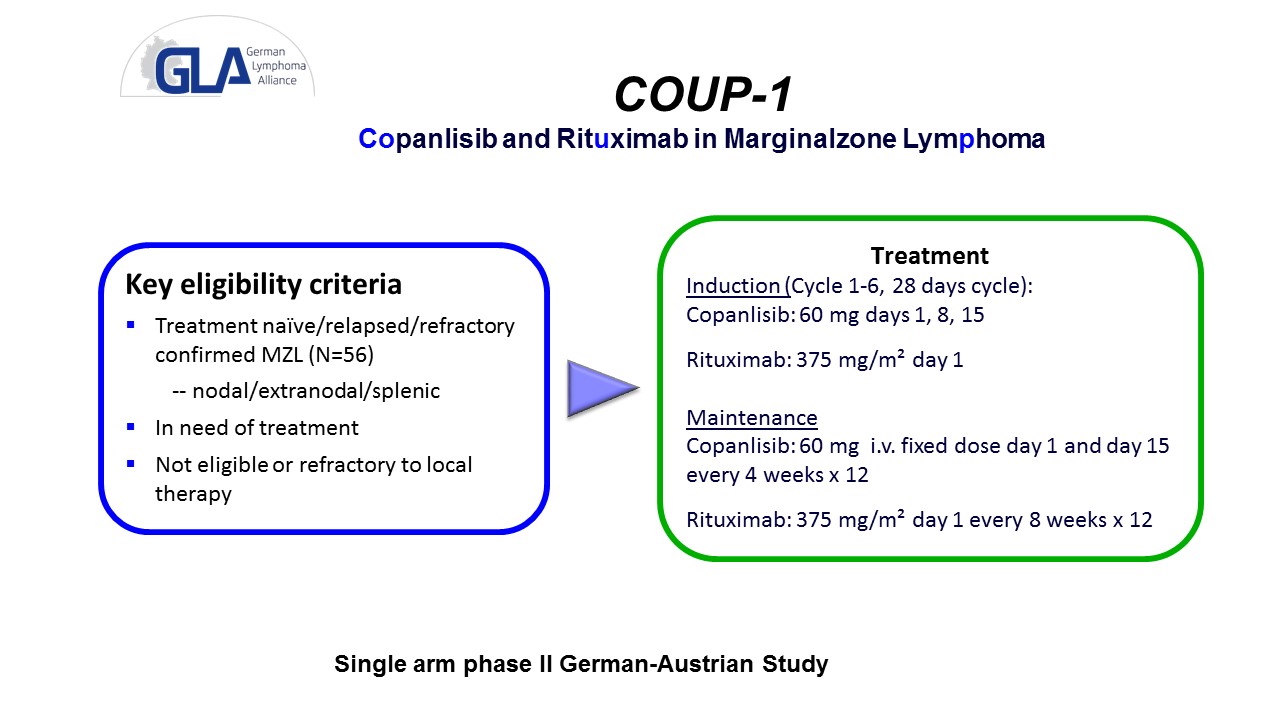 | Fig. 2 |
| Sample size | 14 | For sample size calculation the one-sided one sample exact binomial test was used. According to the available data, the CRR for the total group of the different subtypes of MZL must be better than 56% 12 months after start of induction therapy. Based on a CRR for Copanlisib of about 75%, a significance level of 2.5% (because of one- sided test) and a power of 80%, 48 full evaluable patients will be necessary to show that the combination will be a promising candidate for challenging immuno-chemotherapy (PROC POWER, SAS 9.3; 48 patients is the first number of patients with a stable power of more than 80%). It is expected, that the rate of withdrawal in the study is smaller than 15%. According to these parameters, the study will enrol 56 subjects. The [distribution](http://dict.leo.org/ende?lp=ende&p=thMx..&search=distribution) [between](http://dict.leo.org/ende?lp=ende&p=thMx..&search=between) [the](http://dict.leo.org/ende?lp=ende&p=thMx..&search=the) [sexes](http://dict.leo.org/ende?lp=ende&p=thMx..&search=sexes) is not relevant, because neither incidence of MZL differed between sexes nor clinically outcome measures such as response, progression free and overall survival has been shown to be related to sexes. | 9-10 |
| Recruitment | 15 | approximately 20 investigator sites in Germany and 1 major site in Austria which is supposed to recruit 16 patients | n.a. |
| **Methods: Assignment of interventions (for controlled trials)** | | |  |
| Allocation: |  |  |  |
| Sequence generation | 16a | n.a., no randomization | n.a. |
| Allocation concealment mechanism | 16b | n.a., no randomization | n.a. |
| Implementation | 16c | n.a., no randomization | n.a. |
| Blinding (masking) | 17a | n.a., no blinded treatment | n.a. |
|  | 17b | n.a., no blinded treatment | n.a. |
| **Methods: Data collection, management, and analysis** | | |  |
| Data collection methods,  Data management | 18a, 18b and 19 | An eCRF is set-up by the data management of a CRO and will be completed for each study patient. It is the responsibility of the investigator to ensure the accuracy, completeness, legibility and timeliness of the data reported in the patient’s eCRF which have been designed to record all observations and other pertinent data to the clinical investigation.  Source documentation supporting the eCRF data should indicate the patient’s participation in the study and should document the dates and details of study procedures, adverse events and patient status. Regardless, there must be a minimum documentation, which provides information on study participation and includes all medical information necessary for appropriate medical care outside of the clinical trial in the patient record. In addition, source documents must mention that the patient has been included in an investigational study. Finally, there must be no data that are inconsistent between eCRF and source documents. The investigator is responsible for ensuring that all sections of the eCRF are completed correctly and that entries can be verified against source data. Investigators and site staff will be trained on the eCRF before their account is activated and the can capture data. The investigator, or designated representative, should complete the eCRF pages as soon as possible after information is collected, preferably on the same day that a study patient is seen for an examination, treatment, or any other study procedure. Any outstanding entries must be completed immediately after the final examination. An explanation should be given for all missing data. The correctness of entries in the eCRF will be confirmed by dated electronic signature of the responsible investigator.  Data Handling  To ensure data quality during data capture, the data management has programmed edit checks that pop up while entering data, in case values are out of range or doubtful or missing. After the sign-off of the responsible investigator the data management will check completeness, validity, and plausibility of data by validation programs, which will generate queries. All missing data or inconsistencies will be reported back to the investigators’ sites and have to be clarified by the responsible investigator in due time and prior to database lock. If no further corrections are to be made in the database. it will be declared locked and used for statistical analysis. All data management activities will be done according to a data management plan and the current SOPs of the data management.  All data entry and corrections are recorded in the audit trail (date of data entry/correction, name of person, type of action). | n.a. |
| Statistical methods | 20a-c | The primary endpoint (CRR) will be evaluated 12 months after the last recruited patient has started her/his induction treatment. The primary parameter CRR will be evaluated in a modified intention to treat way, which means that all patients for whom the primary endpoint CRR is measured at 12 months after start of induction therapy will be included in the analysis of the primary endpoint. Only patients who withdraw will be excluded (about 15% are expected). The one sample exact binomial test will be used for the analysis of the primary endpoint to test the CRR against the fixed value 56% at the 2.5% significance level (one-sided). Thus, the decision about the new single agent concept will be based on a statistical test of the form: HA: {CRR > 56%} vs. H0: {CRR ≤ 56%}  Thus, claim of success can be done if 36 (75% of 48 patients) or more responders (patients with CR) will be observed. Patients who withdraw will be included in a separate explorative analysis. Additionally, a one-sided 97. 5% confidence interval for CRR will be calculated as an effect estimator. Exploratory use of univariate logistic regression models will be used to investigate the influence of putative risk factors associated with CRR.  Subgroup analyses in the subtype (MALT lymphoma, splenic MZL, nodal MZL) will be performed as further exploratory analyses.  All secondary endpoints will be analyzed exploratory by respective descriptive analysis and 95%- confidence intervals.  Safety evaluations include: adverse events, vital signs, physical examinations, evaluation of changes to concomitant medications, and clinical laboratory parameters. The severity of adverse events will be assessed using National Cancer Institute Common Terminology Criteria for Adverse Events, Version 5.0. Serious adverse events will be reported according to the GCP regulations. | 9-10 |
| **Methods: Monitoring** | | |  |
| Data monitoring  Harms,  Auditing | 21a-b,  22,  23 | An independent external Data Safety Monitoring Committee (DSMC) will review ongoing safety data throughout the study. The Data Safety Monitoring Board will include at least three independent members (2 experts in MZL and one independent statistician) and the implementation of the DSMC follows strictly the EMA guidelines. (The guidelines may be found at <https://www.ema.europa.eu/documents/scientific-guideline/guideline-data-monitoring-committees_en.pdf>). The DSMC will review data regarding safety as planned according to the DSMC Charter latest 4 weeks after the 6th patient has ended induction and after the 28^th^ patient has ended induction. All data presented at the meeting will be considered confidential. In addition, a review will be performed, when the last patient has ended treatment. Following each meeting the DSMC will prepare a report and may recommend changes in the conduct of the trial. Details on the work of the board will be described in a specific DSMC charter, to be jointly agreed upon the board and the sponsor.  The DMC will get the annual DSURs. The members of the DMC are listed in the protocol. No interim analysis is planned. The trial can be stopped by the LKP if the DMC detects an unexpected accumulation of side effects, has new information about the effect-risk ratio of the investigated therapy or the used methods of examination. The trial can also be stopped by the LKP if recruitment is below the expectations and cannot be improved. All investigators have to be informed immediately about a stopping or a permanent ending of the trial. The participating sites must accept the decision. A participating site can also be closed early by the LKP if the site does not act according to ICH-GCP or according to the trial protocol or the recruitment or the quality of the data is below the expectations. If the DMC suggests an interruption or an ending of the trial due to SAE evaluations or other reasons, the ethical board and the authorities (BOB and sate authority) have to be informed. Monitoring will be done by on-site and remote visits by a clinical monitor according to a Monitoring Plan. The monitor will review the entries into the eCRFs on the basis of source documents. The investigator must allow the monitor to verify all essential documents and must provide support at all times to the monitor. The monitor will document the visit in a report for the sponsor. The site will be provided with a follow-up letter of the findings and the necessary actions to be taken. By frequent communications (letters, telephone, e-mail), the site monitor will ensure that the trial is conducted according to the protocol and regulatory requirements. Frequency and details of monitoring will be defined in a Monitoring Plan. If there are major findings during monitoring or an audit, the investigational site might be closed by the LKP | 9 |
| Ethics and dissemination | | |  |
| Research ethics approval | 24 | The procedures set out in this trial protocol, pertaining to the conduct, evaluation, and documentation of this trial, are designed to ensure that all persons involved in the trial abide by ICH harmonised tripartite guideline on Good Clinical Practice (ICH-GCP) and the ethical principles described in the applicable version of the Declaration of Helsinki. The trial will be carried out in keeping with local legal and regulatory requirements. The regulations of the AMG and GCP regulations as well as the EU Datenschutz-Grundverordnung (DSGVO) will be respected. Before the start of the trial, the trial protocol, informed consent document, and any other appropriate documents have been submitted to the independent country central Ethics Committees (EC) as well as to the competent authorities. A written favourable vote of the ECs and an (implicit) approval by the competent authorities was a prerequisite for initiation of this clinical trial. | 15 |
| Protocol amendments | 25 | The Coordinating Investigators and all investigators will be given an up-to-date investigator’s brochure containing full details of the status of the pre-clinical and clinical knowledge of the study medication. As soon as new information is obtained, an updated version will be supplied or an amendment added to the existing investigator’s brochure. The investigator ensures that all team members are informed adequately about the protocol, all amendments to the protocol, the study procedures und study specific duties and tasks. The investigator will maintain a list to delegate tasks to the team members. | n.a. |
| Consent or assent | 26a | Local physician | n.a. |
|  | 26b | Transfer agreement for optional biosampling may be obtained with the informed consent or assent by the local physician. | n.a. |
| Confidentiality | 27 | The data obtained in the course of the trial will be treated pursuant to the Data Protection Regulation (Datenschutzgrundverordnung, DSGVO). During the clinical trial, subjects will be identified solely by means of their individual identification code. Trial data stored on a computer will be stored in accordance with local data protection law and will be handled in strictest confidence. Distribution of these data to unauthorised persons has to be prevented strictly. The appropriate regulations of local data legislation will be fulfilled in its entirety. The subject consents in writing to release the investigator from his/her professional discretion in so far as to allow inspection of original data for monitoring purposes by health authorities and authorised persons (inspectors, clinical monitors, auditors). Authorised persons (inspectors, clinical monitors, auditors) may inspect the subject-related data collected during the trial ensuring the data protection law. The investigator will maintain a subject identification list (subject numbers with the corresponding subject names) to enable records to be identified. Subjects who did not consent to circulate their pseudonymised data will not be included into the trial. | n.a. |
| Declaration of interests | 28 | The trial is partially supported by Bayer AG. Support includes the supply of the study medication. Also Celltrion Healthcare supplies study medication. There is no other funding of the trial. The authors have no other direct financial relationship with Bayer AG or Celltrion Healthcare. | 14 |
| Access to data | 29 | According to ICH-GCP the investigator(s)/institution(s) must provide direct access to source data/documents for trial related monitoring, audits and regulatory inspection. Each subject has consented - via written informed consent - to direct access to his/her original medical records for trial related monitoring, audit and regulatory inspection. Any data to be recorded directly in the eCRFs (i.e., no prior written or electronic record of data), and to be considered to be source data must be clearly identified. In the absence of either an audit-trail or limited access for the monitor the electronic record of data must be printed out (certified copy). In case of changes a new print-out has to be made. Regulatory authorities and/or auditors authorised by the sponsor may request access to all source documents, eCRFs, and other trial documentation. The investigator who must provide support at all times for these activities must guarantee direct access to these documents. The investigator will inform the sponsor immediately about a planned inspection |  |
| Ancillary and post-trial care | 30 | n.a. | n.a. |
| Dissemination policy | 31a | n.a. | n.a. |
|  | 31b | Publication will be prepared under the lead of the principal investigator of the study. The first and last authorship are reserved for the principal investigator and the study coordinator of the study if both do not wish to transfer their authorship to a third person. All data will be published independently of the results of the trial. | n.a. |
|  | 31c | n.a. | n.a. |
| Appendices |  |  |  |
| Informed consent materials | 32 | Before being admitted to the clinical trial, the patient must consent to participate after being fully informed by the investigator or a designated member of the investigating team about the nature, importance, risks and individual consequences of the clinical trial and their right, to terminate the participation at any time. The patient should also have the opportunity to consult the investigator, or a physician member of the investigating team about the details of the clinical trial. The informed consent to participate in the clinical trial may be withdrawn by the patient verbally in the presence of, or in written form directed to, the investigator or a physician member of the investigating team at any time during the trial. The patient must not entail any disadvantage therefore or be coerced or unduly influenced to continue to participate. Furthermore, the patient is not obligated to disclose reasons for the withdrawal of the consent. If the patient has a primary physician, the investigator should inform him or her about the patient’s participation in the trial, provided the patient agrees hereto. After reading the informed consent document, the patient must give consent in writing. The patient's consent must be confirmed by the personally dated signature of the patient and by the personally dated signature of the physician conducting the informed consent discussion. If the patient is unable to write, oral presentation and explanation of the content of the informed consent form and of the data protection information must take place in the presence of an impartial witness. The witness and the physician conducting the informed consent discussions must also sign and personally date the consent document. The witness must not be in any way dependent on the sponsor of the trial, the trial site or any member of the investigating team (e. g. an employee at the trial site.). A copy of the signed informed consent document must be given to the subject; the original will be filed by the investigator. The documents must be in a language understandable to the subject and must specify who informed the subject. The subjects will be informed as soon as possible if new information may influence his/her decision to participate in the trial. The communication of this information should be documented. The informed consent for genetic profiling and the extended follow-up according to a patient register will be independent of the consent of participation in the trial | 7 |
| Biological specimens | 33 | In the case of patient’s consent, the following biological samples will be obtained: 15 mL bone marrow cells, cheek swab, 50 mL peripheral blood and serum. Six eligible time points are described for bio-sampling: prior to treatment, after end of Induction and before start of Maintenance, Maintenance: Primary endpoint month 12, Maintenance: End of Copanlisib treatment month 20, Maintenance Completion Visit (MCV, 110 days after last dose), at progress.  All samples will be pseudonymized using the patient ID code and send to the Institute of Experimental Cancer Research. All investigations should serve the increase of knowledge about origin, expansion and therapy of indolent lymphomas. There might be new scientific questions/methods concerning the aim of the study, which cannot be addressed today. | 11 |

*It is strongly recommended that this checklist be read in conjunction with the SPIRIT 2013 Explanation & Elaboration for important clarification on the items. Amendments to the protocol should be tracked and dated. The SPIRIT checklist is copyrighted by the SPIRIT Group under the Creative Commons “[Attribution-NonCommercial-NoDerivs 3.0 Unported](http://www.creativecommons.org/licenses/by-nc-nd/3.0/)” license.
