# Supplementary material for: A phase II study of the PI3K inhibitor copanlisib in combination with the anti-CD20 monoclonal antibody rituximab for patients with marginal zone lymphoma: treatment rationale and protocol design of the COUP-1 trial
Source: BMC Cancer. 2021 Jun 29;21:749. doi: 10.1186/s12885-021-08464-6 (PMC8243426; doi:10.1186/s12885-021-08464-6)
Supplement: Supplementary file 2 — Additional file 2. Schedule of enrolment, interventions, and assessments. [file 12885_2021_8464_MOESM2_ESM.docx]

## Additional File II: Schedule of enrolment, interventions, and assessments

**Study Flowchart: Induction treatment**

|  | Screening | Treatment Period**^n^** | | | | | | | | Induction Completion  (1 month after start of cycle 6) / ET Visit |
| --- | --- | --- | --- | --- | --- | --- | --- | --- | --- | --- |
|  | SD  -28 | C1, D1  (SD1)  Month 1 | C1, D8 | C1, D15 | C2, D1,8,15  Month 2 | C3,  D1, 8,15  Month 3 | C4, D1,8,15  Month 4 | C5, D1,8,15  Month 5 | C6, D1,8,15  Month 6 |  |
| Informed consent | X |  |  |  |  |  |  |  |  |  |
| Discussion of fertility issues and sperm banking (in patients of childbearing age) | X |  |  |  |  |  |  |  |  |  |
| Inclusion/exclusion criteria | X | X |  |  |  |  |  |  |  |  |
| Pathological diagnosis (local and reference pathology – incl. MYD88) | X |  |  |  |  |  |  |  |  |  |
| Demographics and medical history^a^ | X | X |  |  |  |  |  |  |  |  |
| Gastroduodenal endoscopy^b^ | X |  |  |  |  |  | X (d1) |  |  | X |
| Prognostic Score for extranodal MZL (MALT-IPI) | X |  |  |  |  |  |  |  |  |  |
| Physical examination ^c^ | X | X | X | X | X | X | X | X | X | X |
| Vital signs^d^ | X | X | X | X | X | X | X | X | X | X |
| ECOG Performance Status | X |  |  |  | X | X | X | X | X | X |
| B symptoms | X | X | X | X | X | X | X | X | X | X |
| 12-lead ECG | X |  |  |  |  |  |  |  |  | X |
| Echocardiography | X |  |  |  |  |  |  |  |  | X |
| Hematology | X | X | X | X | X | X | X | X | X | X |
| Serum chemistry and electrophoresis^e^ | X | X | X | X | X | X (d1) | X (d1) | X (d1) | X (d1) | X |

|  | Screening | Treatment Period**^n^** | | | | | | | | Induction Completion  (1 month after start of cycle 6) / ET Visit |
| --- | --- | --- | --- | --- | --- | --- | --- | --- | --- | --- |
|  | SD  -28 | C1, D1  (SD1)  Month 1 | C1, D8 | C1, D15 | C2, D1,8,15  Month 2 | C3,  D1, 8,15  Month 3 | C4, D1,8,15  Month 4 | C5, D1,8,15  Month 5 | C6, D1,8,15  Month 6 |  |
| Hemoglobin A1c^f^ | X |  |  |  |  |  | X (d1) |  |  | X |
| Glucose | X | X | X | X | X | X | X | X | X |  |
| Coagulation (PTT, PT, INR) | X | X |  |  |  |  |  |  |  | X |
| HIV, HBC, HCV serology | X |  |  |  |  |  |  |  |  |  |
| HBV DNA PCR | (X) |  |  |  | (X) | (X) | (X) | (X) | (X) | (X) |
| Urine analysis^g^ | X |  |  |  | X (d1) | X (d1) | X (d1) | X (d1) | X (d1) | X |
| Pregnancy test | X | X |  |  | X (d1) | X (d1) | X (d1) | X (d1) | X (d1) | X |
| New anti-lymphoma treatment |  |  |  |  |  |  |  |  |  | X |
| CT/MRI scan and tumor assessment | X |  |  |  |  |  | X^k^ (d1) |  |  | X |
| Bone marrow aspirate and biopsy | X^l^ |  |  |  |  |  |  |  |  | X^o^ |
| Leukocyte immunophenotyping (FACS) PB and BM | X |  |  |  |  |  | X^h^ (d1) (PB only) |  |  | X^h^ |
| Quantitative immunoglobulins (IgA, IgG, IgM) | X |  |  |  |  |  | X (d1) |  |  | X |
| Serum protein immunofixation | X |  |  |  |  |  | X^i^ (d1) |  |  | X^i^ |
| Tumor tissue sample | X | (X)^j^ | | | | | | | | |
| (S)AE | X | X | X | X | X | X | X | X | X | X |
| Concomitant medication | X | X | X | X | X | X | X | X | X | X |
| QoL questionnaire (FACT-Lym) |  | X |  |  |  | X (d1) |  |  |  | X |
| Bone marrow cells storage, DNA and RNA^m^ | X |  |  |  |  |  |  |  |  | X^o^ |
| Blood cells, serum plasma storage^m^ | X |  |  |  |  |  |  |  |  | X |

AE=adverse event; BM=bone marrow; C=Cycle; CR=complete response; CT=computed tomography; D=Day; ECG=electrocardiogram; ECOG=Eastern Cooperative Oncology Group; ET=early termination; FACS=fluorescence-activated cell sorter; HBcAb=hepatitis B core antibody; HBsAb=hepatitis B surface antibody; HBsAg=hepatitis B surface antigen; HBV=hepatitis B virus; HCV=hepatitis C virus; INR=international normalized ratio; IPI=International Prognostic Index; IV=intravenous; LDH=lactate dehydrogenase; MALT=mucosa-associated lymphatic tissue; MRI=magnetic resonance imaging; PB=peripheral bloodPR=partial response; PRO=patient-reported outcome; SAE=serious adverse event; SD=Study Day.

Notes: On treatment days, all assessments should be performed prior to dosing unless otherwise noted. The C1, D1 visit should be scheduled to allow subsequent visits to occur without delay. Study visits during treatment should occur on the scheduled day (±4 days), with the exception of delays resulting from toxicities.

(x)=conditional/optional; refer to footnote for details.

^a^ At screening, obtain demographic data and complete medical history. On C1, D1 obtain medical history since the screening visit.

^b^ In patients with gastric extranodal MZL.

^c^ At screening a complete physical examination will be performed. At the other visits physical examination should only include systems of most clinical relevance.

^d^ Includes blood pressure, pulse rate, and temperature.

^e^ β_2_ microglobulin is required only at screening. Cryoglobulins, cold agglutinins and Coombs-Test should also be determined at screening and repeated if initially positive, at least at C4D1 and Induction Completion and when clinically indicated.

^f^ Hemoglobin A1c (HbA1c) at Screening, on Day 1 of every three cycles (4, 7, 10, etc.) and at the EOT visit. The testing is not required if the previous test was performed within 4 weeks preceding EOT visit.

^g^ If initially positive for proteinuria

^h^ A blood sample will be taken for the assessment of circulating lymphoma cells: mandatory: CD5−, CD10−, CD19+, CD23−, CD27+, CD43−, FMC7±, kappa/lambda. BM immunophenotyping only at Induction Completion and if initially positive.

^i^ If initially positive for monoclonal Ig

^j^ If an additional biopsy is done as part of the standard of care at the time of relapse or transformation, a tissue sample will be collected at that time.

^k^ Mandatory for patients with nodal, splenic and extragastric MZL. For patients with gastric MZL only if there are extragastric manifestation.

**^l^** acceptable up to 12 weeks before start of treatment

^m^ optional Biosampling

**^n^** The first 6 included patients will be closely safety monitored. For these 6 patients safety evaluations (vital signs, physical examination, hematology, serum chemistry and coagulation assessments) will be performed every week for cycle 1 (i.e. also on Day 22) and week 1 and 3 for cycle 2-6, i.e. Day 1 and Day 15.

^o^ if required to confirm a CR and if clinically indicated

**Study Flowchart: Maintenance**

| Months from last treatment cycle |  | | Maintenance Treatment | | | | | | | | | | | | | | | | | | | | | | | | | | | | | | | | | Maintenance Completion/  ET Visit |
| --- | --- | --- | --- | --- | --- | --- | --- | --- | --- | --- | --- | --- | --- | --- | --- | --- | --- | --- | --- | --- | --- | --- | --- | --- | --- | --- | --- | --- | --- | --- | --- | --- | --- | --- | --- | --- |
|  | 2 | | 3 | | 4 | | 5 | | 6  Evaluation of the primary endpoint of the study | | 7 | | 8 | | 9 | | 10 | | 11 | | 12 | | 13 | | 14 | | 16 | | 18 | | 20 | | 22 | | 24 | 110 days after last dose |
| Physical examination ^a^ | X | | X | | X | | X | | X | | X | | X | | X | | X | | X | | X | | X | | X | | X | | X | | X | | X | | X | X |
| Vital signs ^b^ | X | | X | | X | | X | | X | | X | | X | | X | | X | | X | | X | | X | | X | | X | | X | | X | | X | | X | X |
| ECOG Performance Status | X | | X | | X | | X | | X | | X | | X | | X | | X | | X | | X | | X | | X | | X | | X | | X | | X | | X | X |
| B symptoms | X | | X | | X | | X | | X | | X | | X | | X | | X | | X | | X | | X | | X | | X | | X | | X | | X | | X | X |
| Hematology | X | | X | | X | | X | | X | | X | | X | | X | | X | | X | | X | | X | | X | | X | | X | | X | | X | | X | X |
| Serum chemistry | X | | X | | X | | X | | X | | X | | X | | X | | X | | X | | X | | X | | X | | X | | X | | X | | X | | X | X |
| Hemoglobin A1c^c^ |  | |  | | X | |  | |  | | X | |  | |  | | X | |  | |  | | X | |  | | X | |  | |  | |  | |  | X |
| Glucose | X | | X | | X | | X | | X | | X | | X | | X | | X | | X | | X | | X | | X | | X | | X | | X | | X | | X | X |
| Urine analysis^d^ |  | |  | |  | |  | | X | |  | |  | |  | |  | |  | | X | |  | |  | | X | |  | |  | |  | | X | X |
| Pregnancy test | X | | X | | X | | X | | X | | X | | X | | X | | X | | X | | X | | X | | X | | X | | X | | X | | X | | X | X |
| HBV DNA PCR^e^ | (X) | | (X) | | (X) | | (X) | | (X) | | (X) | | (X) | | (X) | | (X) | | (X) | | (X) | | (X) | | (X) | | (X) | | (X) | | (X) | | (X) | | (X) | (X) |
| New anti-lymphoma treatment | X | | X | | X | | X | | X | | X | | X | | X | | X | | X | | X | | X | | X | | X | | X | | X | | X | | X | X |
| CT/MRI scan and tumor assessment (if initially positive) |  | |  | |  | |  | | X | |  | |  | |  | | X | |  | |  | |  | | X | |  | |  | | X | |  | |  | X |
| Months from last treatment cycle | |  | | Maintenance Treatment | | | | | | | | | | | | | | | | | | | | | | | | | | | | | | | | Maintenance Completion/  ET Visit |
|  |  | 2 | | 3 | | 4 | | 5 | | 6  Evaluation of the primary endpoint of the study | | 7 | | 8 | | 9 | | 10 | | 11 | | 12 | | 13 | | 14 | | 16 | | 18 | | 20 | | 22 | 24 | 110 days after last dose |
| Bone marrow aspirate and biopsy^h^ | |  | |  | |  | |  | | X | |  | |  | |  | | X | |  | |  | |  | | X | |  | |  | | X | |  |  | X |
| Gastroduodenal endoscopy^f^ | |  | |  | |  | |  | | X | |  | |  | |  | | X | |  | |  | |  | | X | |  | | X | |  | | X |  | X |
| Leukocyte immunophenotyping (FACS)^g^ | |  | |  | |  | |  | | X | |  | |  | |  | | X | |  | |  | |  | | X | |  | | X | |  | | X |  | X |
| Quantitative immunoglobulins (IgA, IgG, IgM)^k^ | |  | |  | |  | |  | | X | |  | |  | |  | | X | |  | |  | |  | | X | |  | | X | |  | | X |  | X |
| Serum protein immunofixation (if initially positive) | |  | |  | |  | |  | | X | |  | |  | |  | | X | |  | |  | |  | | X | |  | | X | |  | | X |  | X |
| Tumor tissue sample | | (X)^i^ | | | | | | | | | | | | | | | | | | | | | | | | | | | | | | | | | | |
| (S)AE | | X | | X | | X | | X | | X | | X | | X | | X | | X | | X | | X | | X | | X | | X | | X | | X | | X | X | X |
| Concomitant medications | | X | | X | | X | | X | | X | | X | | X | | X | | X | | X | | X | | X | | X | | X | | X | | X | | X | X | X |
| PRO questionnaire (FACT-Lym) | |  | |  | |  | |  | | X | |  | |  | |  | |  | |  | | X | |  | |  | |  | |  | |  | |  |  | X |
| Bone marrow cells storage, DNA and RNA^j^ | |  | |  | |  | |  | | X | |  | |  | |  | |  | |  | |  | |  | | X | |  | |  | |  | |  |  | X |
| Blood cells, serum plasma storage^j^ | |  | |  | |  | |  | | X | |  | |  | |  | |  | |  | |  | |  | | X | |  | |  | |  | |  |  | X |

AE=adverse event; Cycle; CT=computed tomography; D=Day; ECOG=Eastern Cooperative Oncology Group; ET=early termination; FACS=fluorescence-activated cell sorter; FACT-Lym=Functional Assessment of Cancer Therapy for Lymphoma; HBcAb=hepatitis B core antibody; HBsAg=hepatitis B surface antigen; HBV=hepatitis B virus; LDH=lactate dehydrogenase; MRI=magnetic resonance imaging; PRO=patient-reported outcome; SAE=serious adverse event. Notes: On treatment days, all assessments should be performed prior to dosing unless otherwise noted. Study visits should occur on the scheduled day ±7 days in the first year and ±14 days in the second year, with the exception of delays resulting from toxicities. (x)=conditional/optional; refer to footnote for details.

^a^ Physical examination should only include systems of most clinical relevance.

^b^ Includes blood pressure, pulse rate, and temperature.

^c^ Hemoglobin A1c (HbA1c) will be tested in the maintenance at month 4, 7, 10, 13, 16.

^d^ If initially positive for proteinuria

^e^ Performed for patients who are HBsAg negative and HBcAb positive at screening only. HBV DNA levels will be measured by real-time PCR using an assay with a sensitivity of at least 10 IU/mL.

^f^ In patients with gastric extranodal MZL. If clinically indicated it may be performed even more often.

^g^ A blood sample will be taken for the assessment of circulating lymphoma cells: mandatory: CD5−, CD10−, CD19+, CD23−, CD27+, CD43−, FMC7±, kappa/lambda.

^h^ If initially positive and for confirmation of a CR

^i^ If an additional biopsy is done as part of the standard of care at the time of relapse or transformation, a tissue sample will be collected at that time.

^j^ optional biosampling (if clinically indicated)

^k^ Cryoglobulins, cold agglutinins and Coombs-Test should also be determined and repeated if initially positive at screening.

**Study Flowchart: Follow-up**

| After last treatment cycle or after early termination | Follow-up Year 1  (every 3 months ± 14 days) | | | | Follow-up Year 2  (every 3 months ± 14 days) | | | | Follow-up Year 3  (every 6 months ± 14 days) | | Follow-up Year 4  (every 6 months ± 14 days) | | Follow-up Year 5  (every 6 months ± 14 days) | | At the end of study | At time of progression^d^ |
| --- | --- | --- | --- | --- | --- | --- | --- | --- | --- | --- | --- | --- | --- | --- | --- | --- |
|  | FU1 | FU2 | FU3 | FU4 | FU5 | FU6 | FU7 | FU8 | FU9 | FU10 | FU11 | FU12 | FU13 | FU14 |  |  |
| Physical examination^a^ | X | X | X | X | X | X | X | X | X | X | X | X | X | X | X | X |
| ECOG Performance Status |  | X |  | X |  | X |  | X | X | X |  | X |  | X | X | X |
| B symptoms | X | X | X | X | X | X | X | X | X | X | X | X | X | X | X | X |
| Hematology | X | X | X | X | X | X | X | X | X | X | X | X | X | X | X | X |
| Serum chemistry | X | X | X | X | X | X | X | X | X | X | X | X | X | X | X | X |
| Urine analysis^b^ |  | X |  | X |  | X |  | X | X | X |  | X |  | X | X | X |
| HBV DNA PCR^c^ | (X) | (X) | (X) | (X) |  |  |  |  |  |  |  |  |  |  |  |  |
| New anti-lymphoma treatment^d^ | X | X | X | X | X | X | X | X | X | X | X | X | X | X | X | X |
| Survival follow-up^d^ | X | X | X | X | X | X | X | X | X | X | X | X | X | X | X | X |
| CT/MRI scan and tumor assessment (if initially positive) |  | X |  | X |  | X |  | X | X | X |  | X |  | X | X | X |
| Gastroduodenal endoscopy^e^ |  | X |  | X |  | X |  | X | X | X |  | X |  | X | X | X |
| Leukocyte immunophenotyping (FACS)^f^ |  | X |  | X |  | X |  | X |  | X |  |  |  |  | X | X |
| Quantitative immunoglobulins (IgA, IgG, IgM)^j^ |  | X |  | X |  | X |  | X |  | X |  |  |  |  | X | X |
| Serum protein immunofixation (if initially positive) |  | X |  | X |  | X |  | X |  | X |  |  |  |  | X | X |
| Bone marrow aspirate and biopsy^g^ |  |  |  |  |  |  |  |  |  |  |  |  |  |  | X | X |
| Tumor tissue sample | (X)^h^ | | | | | | | | | | | | | | | |
| PRO questionnaire (FACT-Lym) |  |  |  | X |  |  |  | X |  | X |  | X |  | X | X | X |
| Bone marrow cells storage, DNA and RNA^i^ |  |  |  |  |  |  |  |  |  |  |  |  |  |  |  | X |
| Blood cells, serum plasma storage^i^ |  |  |  |  |  |  |  |  |  |  |  |  |  |  |  | X |

CT=computed tomography; ECOG=Eastern Cooperative Oncology Group; FACS=fluorescence-activated cell sorter; FACT-Lym=Functional Assessment of Cancer Therapy for Lymphoma; HBV=hepatitis B virus; MRI=magnetic resonance imaging; PRO=patient-reported outcome;

(x)=conditional/optional; refer to footnote for details.

^a^ Physical examination should only include systems of most clinical relevance.

^b^ If initially positive for proteinuria.

^c^ Only if clinically indicated. HBV DNA levels will be measured by real-time PCR using an assay with a sensitivity of at least 10 IU/mL.

^d^ Patients with disease progression will be followed for new anti-lymphoma treatment and survival every six months until the end of the study (maximum of 10 years after inclusion of first patient).

^e^ In patients with gastric extranodal MZL. If clinically indicated it may be performed even more often.

^f^ A blood sample will be taken for the assessment of circulating lymphoma cells: mandatory: CD5−, CD10−, CD19+, CD23−, CD27+, CD43−, FMC7±, kappa/lambda

^g^ If initially positive or clinically indicated or in the case of progression

^h^ If an additional biopsy is done as part of the standard of care at the time of relapse or transformation, a tissue sample will be collected at that time.

^i^ optional Biosampling

^j^ Cryoglobulins, cold agglutinins and Coombs-Test should also be determined and repeated if initially positive at screening.
